# Food allergy and anaphylaxis – 2062. “Allergic gastroenteropathy” in UK children

**DOI:** 10.1186/1939-4551-6-S1-P145

**Published:** 2013-04-23

**Authors:** Kirn Sandhu, Karen Adams, Than Soe, Janice Mccreadie, Vijay Chakravarti

**Affiliations:** 1 Child Health, Princess Alexandra Hospital, Harlow, UK

## Background

To establish the clinical spectrum of food related (allergic) gastroenteropathy in children.

## Methods

30 children with allergic bowel disease were identified from general and paediatric allergy outpatient clinics and studied retrospectively from the bowel as well as other allergy and management point of view.

## Results

24 of the children (80%) exhibited symptoms of gastro-oesophageal reflux disease, 28 children (93%) had lower GI symptoms and 23 children (77%) had significant sleep problems. 19 out f the 30 children (63%) had other atopic disorders. The most common food trigger was cow’s milk others included egg, soya, wheat, fish, and nuts. Family history of atopy was seen in 24 children (80%). Skin prick tests were positive in only 7 children (23%) suggesting this is a non-IgE mediated phenomenon. Management of these children included diet exclusion, anti-reflux and anti-allergy medication. 12 children (44%) responded poorly to treatment and required steroids and 8 (30%) required biopsies. In families with more than one child with allergic gastroenteropathy, diagnosis and treatment with anti-reflux, diet exclusion and anti-allergy medication was started much earlier (2m-2yrs) in the younger sibling.

## Conclusions

In children the triad of upper and lower GI symptoms, other atopic disorder and a strong family history of atopy suggests a diagnosis of allergic bowel disease (allergic gastroenteropathy). This condition is diagnosed commonly as Gastroesophageal reflux disease with milk allergy but this fails to recognise the significant mid and lower bowel component in most of these children, which can be considered as an allergic inflammatory bowel disease. It is neither necessary nor essential to biopsy all these children in view of the response to anti allergy and anti inflammatory measures as well as the large number of children with this problem. Biopsies when positive may have esoinophils, hence this may represent the milder end of the spectrum of eosinophilic gastrointestinal disorder (EGID). The management of allergic gastroenteropathy ranges from dietary exclusion, anti-reflux & anti-allergy medication to immunomodulators in severe cases.

